# Bondline Thickness Effects on Damage Tolerance of Adhesive Joints Subjected to Localized Impact Damages: Application to Leading Edge of Wind Turbine Blades

**DOI:** 10.3390/ma14247526

**Published:** 2021-12-08

**Authors:** Amrit Shankar Verma, Nils Petter Vedvik, Zhen Gao, Saullo G. P. Castro, Julie J. E. Teuwen

**Affiliations:** 1 Department of Mechanical Engineering, The University of Maine, Orono, ME 04473, USA; 2 Department of Mechanical and Industrial Engineering, Norwegian University of Science and Technology (NTNU), 7491 Trondheim, Norway; nils.p.vedvik@ntnu.no; 3 Department of Marine Technology, Norwegian University of Science and Technology (NTNU), 7491 Trondheim, Norway; zhen.gao@ntnu.no; 4 Faculty of Aerospace Engineering, Delft University of Technology (TU Delft), 2628 CD Delft, The Netherlands; S.G.P.Castro@tudelft.nl (S.G.P.C.); J.J.E.Teuwen@tudelft.nl (J.J.E.T.)

**Keywords:** offshore wind turbine, wind turbine blade, impact, damage, composite

## Abstract

The leading edges of wind turbine blades are adhesively bonded composite sections that are susceptible to impact loads during offshore installation. The impact loads can cause localized damages at the leading edges that necessitate damage tolerance assessment. However, owing to the complex material combinations together with varying bondline thicknesses along the leading edges, damage tolerance investigation of blades at full scale is challenging and costly. In the current paper, we design a coupon scale test procedure for investigating bondline thickness effects on damage tolerance of joints after being subjected to localized impact damages. Joints with bondline thicknesses (0.6 mm, 1.6 mm, and 2.6 mm) are subjected to varying level of impact energies (5 J, 10 J, and 15 J), and the dominant failure modes are identified together with analysis of impact kinematics. The damaged joints are further tested under tensile lap shear and their failure loads are compared to the intact values. The results show that for a given impact energy, the largest damage area was obtained for the thickest joint. In addition, the joints with the thinnest bondline thicknesses displayed the highest failure loads post impact, and therefore the greatest damage tolerance. For some of the thin joints, mechanical interlocking effects at the bondline interface increased the failure load of the joints by 20%. All in all, the coupon scale tests indicate no significant reduction in failure loads due to impact, hence contributing to the question of acceptable localized damage, i.e., damage tolerance with respect to static strength of the whole blade.

## 1. Introduction

### 1.1. Background

**Figure 1:**
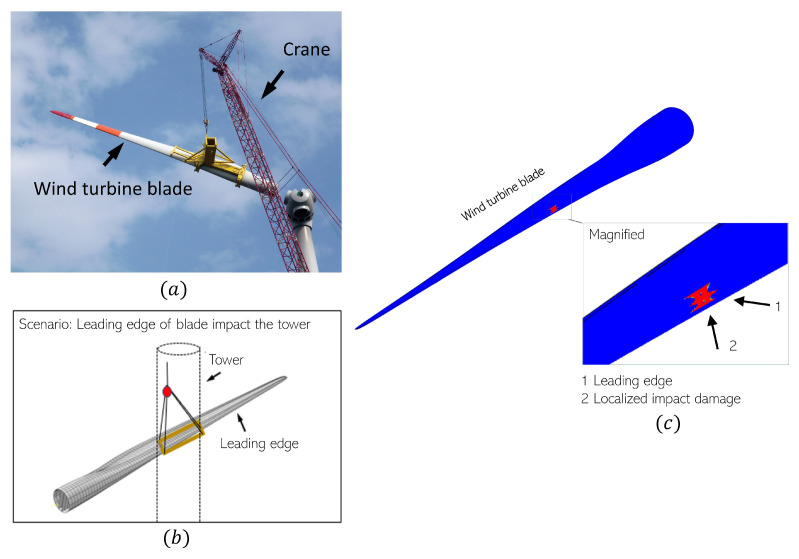
(**a**) Lifing phase of wind turbine blade [5]; (**b**) collision scenario [2]; (**c**) localized impact damage at adhesive joint of leading edge [2].

The recent trends in the offshore wind turbine industry include bigger turbines with large size rotor blades as they increase the rotor swept area and power production, and are thus profitable for the industry [1]. However, the increasing size of wind turbine blades creates complex challenges during their assembly and installation phases in the offshore environment [2,3]. One of the major issues is the risk of impact loads with the surrounding structures due to substantial dynamic motion responses existing during the lifting phase using offshore crane vessels; see Figure 1a. Verma et al. [2] identified different collision scenarios during blade installation. The study found that large pendulum motions during lifting can cause the leading edge of the blade to collide with the preassembled structure, such as the turbine tower (Figure 1b) [4]. Verma et al. [2] numerically investigated this collision scenario and it was found that the blade could hit the turbine tower with low velocity, causing localized damages at the leading edge of the adhesive joint (see Figure 1c). Given that leading edges of wind turbine blades carry complex operational loads during their service life, it becomes extremely crucial to understand the effect of impact-induced local damages on the blade’s damage tolerance. However, performing damage tolerance investigations at full-scale present complex challenges using experimental or numerical procedures. The experimental procedures will involve several parameters such as varying contact regions, varying bondline thicknesses, as well as complex material combinations along the blade’s leading edges. All these parameters invoke several sources of uncertainty and it becomes a demanding task to characterize and study damage tolerance at a full scale. Similarly, a numerical-based technique will require a mesoscale-based modeling procedure, which will be computationally expensive at a full scale. Additionally, the reliability of these numerical models will be questionable as material parameters required for damage models are not always readily available.

Owing to these complex challenges, full scale tests are generally used for certification purposes [6,7]. For detailed analyses, such as impact damages and damage tolerance investigations, a small-scale coupon test program is preferred for composite structures to gain insights into material performance [6]. In addition, numerical models are also built at a coupon scale that can be applied at full scale. With regards to the collision scenario of the blade’s leading edge during installation, we hypothesize that an efficient coupon scale damage tolerance test procedure is required to gain insights into the blade’s damage tolerance at full scale. Note that the use of the word ‘scale’ in the paper is in the context of the scale of analysis performed for composite structures (i.e., coupon scale, full scale, or component scale). In this work, we have designed a novel coupon scale test procedure to investigate bondline thickness effects on damage tolerance of adhesive joint subjected to localized impact damages.

A literature review is presented below that details about the blade’s leading edges including past studies where impact load and impact damage tolerance investigations have been performed.

### 1.2. Literature Review: Wind Turbine Blades, Impact Loads, and Damage Tolerance Investigations

**Figure 2:**
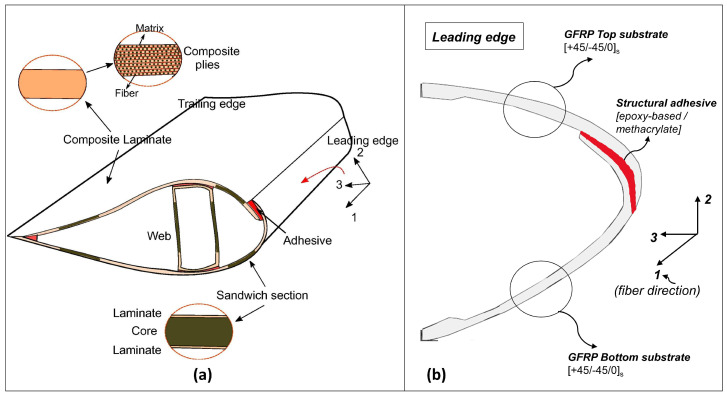
(**a**) Wind turbine blade cross section [9]. (**b**) Leading edge joint with top and bottom substrate with material orientation (${\left[+45/-45/0\right]}_{s}$) (where 0-degree layer is along the blade span).

A typical composition of a wind turbine blade and their leading edges is presented in Figure 2a,b. It can be seen that the leading edges are primarily composed of adhesively bonded composite laminates made of Glass Fiber Reinforced Plastic (GFRP) materials and have plies with layup oriented in (${\left[+45/-45/0\right]}_{s}$) material directions where a 0-degree layer is along the blade span. The main reasons for application of adhesive bonds over mechanical fastenings are their ability to facilitate in uniform stress distributions, and to join large and dissimilar adherends [8]. The adhesive bonds also include other advantages, such as high fatigue strength and corrosion resistances, while maintaining the integrity of the entire blade structure.

Generally, structural adhesives are used for the bonding process in the blade given that they act as load-bearing material and also provide additional strength to the adjoining substrates [10]. There are several varieties of structural adhesives utilized in the blade industry such as epoxy adhesive, vinyl-ester adhesive, and methyl methacrylate adhesive. However, epoxy-based structural adhesives are most commonly used given that they have higher shear strengths compared to other structural adhesives [11].

The impact response of an adhesive joint, e.g., for a wind turbine blade section, will be a combined contribution of the impact response of composite adherend together with structural adhesive material. In general, composite material provides high stiffness to weight ratio and provides sound stiffness and strength in the fiber-axis direction [12]. However, these materials are weak in the thickness direction and can cause complex failure modes under impact loads that interact simultaneously and are visually undetectable [13]. In the literature, there are not many studies where impact loads investigations have been performed specifically for wind turbine blade adhesive sections. Some of the studies include impact load assessments on wind turbine blades due to bird collision [14], high-velocity hail impact [15], rain droplet impact [16], as well as impact with preassembled structures during transportation and installation [3,9,17,18]. In these studies, complex failure modes have been obtained in the blades due to impact [9], which include matrix cracking, delamination of plies, and face core debonding. The delamination of plies in composite structures is not visually detectable, and involves local separation of laminated structures into sublaminates [19]. This causes a reduction in the critical buckling loads, including a reduction in the overall strength and stiffness of the structure. The presence of delamination can be critical for blades as compressive loads are generated on the suction side of blades due to blade bending during operations. Since varying blade regions, such as leading edges, trailing edges, and blade roots are vulnerable to impact loads [2], impact damage tolerance investigations for blades are vital. Haselbach et al. [19] numerically studied the effect of delaminations in the blade spar on the strength of rotor blades. It was found that delamination close to the blade surface leads to local buckling modes and is more critical than delamination near the mid surface in the spar. Other blade-specific damage tolerance studies can be found in [7,20,21,22,23,24]. There are also records of impact damage tolerance studies for thin-walled composite structures applications in other industries, such as aerospace, aircraft, automotive, and civil. For such applications, the damage tolerance investigation involves compression after impact (CAI) studies on standardized coupon scale samples [25,26]. In addition, component level impact damage tolerance investigations have been performed for stiffened composite panels [27,28,29]. Lastly, several studies have been performed on thin-walled channel section profiles [30,31,32,33] that bridge the gap between simplified coupon scale investigations and advanced component level stringer stiffened panels.

On the other hand, not many studies are found that describe adhesive joint damage of blades due to impact loads as well as their impact damage tolerance investigations. Nevertheless, adhesive joints in a wind turbine blade are prone to high static and fatigue-induced stresses owing to complex in-service loading conditions from flapwise and edgewise loads [34]. Debonding of adhesive joints was found as one of the major driving failure modes during operation [35]. A forensic investigation performed in [36] on a damaged section of a blade revealed such joint failure between blade skin and spar. In [37], a full-scale blade testing was performed under the flapwise loading condition, and it was reported that the first failure initiation started at the failure of adhesive bonds in the wind turbine blades. Additionally, failure of adhesive bond causes progressive failure of the wind turbine blade at increased loads. Overall, adhesive joints are critical locations of weakness in a wind turbine blade from a perspective of structural integrity, hence requiring reliable designing and strength predictions. In addition to the complex in-service loading conditions, adhesive joints at the wind turbine blades must also provide resistances against impact loads, and therefore it is necessary to understand the effects of impact-induced localized damage on their failure strength.

Design guidelines from classification societies such as DNV [38] provide recommendations and recognize impact damages on a wind turbine blade as one of the important design parameters. It is recommended that the sensitivity of the Fibre Reinforced Plastic (FRP) structure under impact loads for critical impact scenarios must be determined by experimental tests. The guidelines suggest that the impact test can be performed on two different structural scales—(1) impact assessment on a material, small section, or coupon scale for a given impact scenario, or (2) impact assessment on the entire component level. The guidelines mention selecting the boundary conditions of test setups and geometry of the impactor, which represents the worst case for the impact scenario [38]. Given that there are various parameters that influence the strength of leading edge joints in combination with impact loads [39,40], such as—(a) adhesive bonding process, (b) surface preparation of joints, (c) adhesive and substrate material properties, (d) fiber orientations of the plies, and (e) geometrical parameters such as overlap length and adhesive thickness, the present study develops a coupon scale test procedure to investigate the damage tolerance. Furthermore, one main parameter—thickness of adhesive joints ($t_{a}$), (herein referred to as the bondline thickness)—is given special emphasis to understand the influences of impact loads on the failure strength of adhesive joints, given that the bondline thickness varies significantly in a wind turbine blade [11].

In the present study, we designed a coupon scale test procedure to investigate the effect of bondline thickness on damage tolerance of adhesive joint subjected to localized impact damage. Three different joints with varying bondline thicknesses ($t_{a}=$ 0.6 mm, 1.6 mm, 2.6 mm) are prepared using glass-fiber reinforced plastic (GFRP) substrates with layup ${\left[+45/-45/0\right]}_{s}$, joined by epoxy structural adhesive Araldite 2015-1. A series of experiments are conducted where the joints are subjected to three different impact energies (5 J, 10 J, and 15 J). The damaged adhesive joints are then loaded under lap shear and their failure loads are recorded. The remaining paper is arranged as follows: Section 2 describes the design of experiments. Section 3 presents the methodology section. Section 4 deals with results and discussions. Finally, conclusions from the study are itemized in Section 5.

## 2. Experimental Design

In the present study, we designed a coupon scale test procedure according to guidelines from [38] to investigate the effect of bondline thickness on damage tolerance of adhesive joint subjected to localized impact damage. The coupon scale test procedure should represent the global impact scenario as closely as possible by—(1) representing the blade’s true geometrical parameters like adhesive thickness or composite thickness, joint configuration, and composite layup at coupon scale (2) using appropriate boundary conditions (BCs) to map the energies associated with damages at full scale to damages at coupon scale, and finally (3) utilizing a representative benchmark testing that would match the operational loads at the blade’s leading edge. These are discussed below:

### 2.1. Coupon Scale Representation of Leading Edge and Benchmark Testing

**Figure 3:**
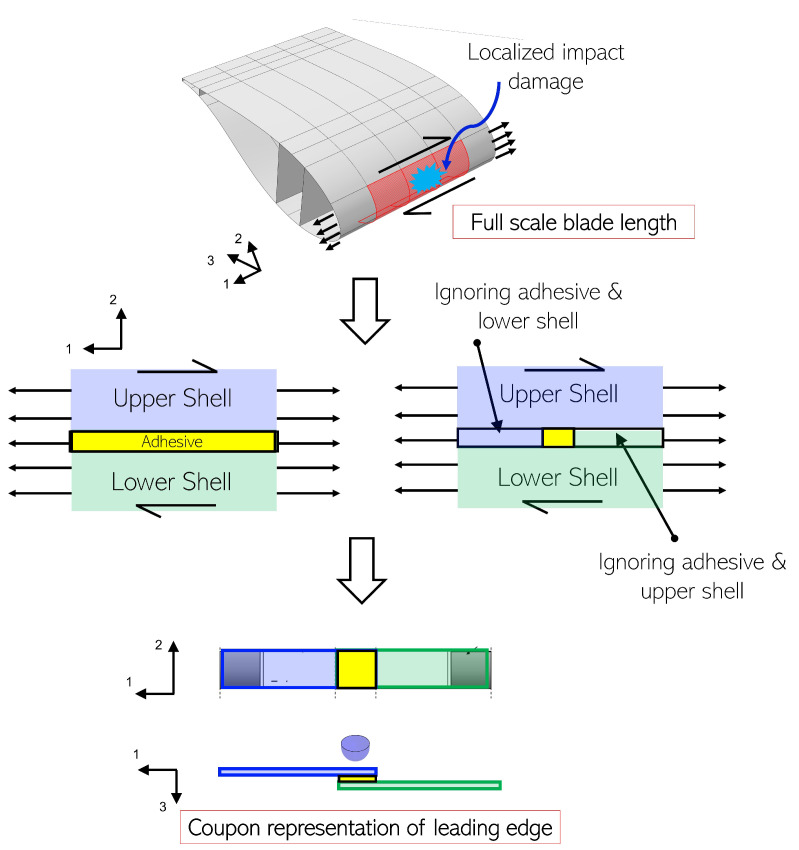
Single lap adhesive joint representation of leading edge at coupon scale.

The leading edge joint connects the upper and lower aerodynamic shells and sometimes a flange is used to redirect the peel stresses to shear stresses [11]. Typical loads on the leading edge of a wind turbine blade consisting of normal force and shear force is shown in Figure 3. Given that the dominant forces are in plane and directed along the adhesive, and can force the substrates to slide over one another, we used the shear mode as the benchmark loading condition and tensile single lap shear tests as benchmark testing for damage tolerance investigation. Note that the tensile single lap shear tests present challenges [41], especially due to paths that create an eccentric load, developing a combination of normal and bending forces in the adhesive zone referred to as peeling forces. Nevertheless, this mode of testing adhesive joints has been used extensively due to simplified geometry to check the performance of the adhesive joints in the state of shear. Furthermore, we represent the leading edge joint by a single lap adhesive joint. Figure 3 describes the assumptions made while making this choice, where we have ignored parts of the adhesive and laminates for our analysis. Similar simplification of leading edge joints has been performed in [42,43]. Note that we represent the blade’s true geometrical parameters like adhesive thickness, composite thickness, joint configuration, and composite layup at coupon scale. These details will further be discussed in Section 3.

### 2.2. Test Setup for Generating Localised Impact Damage

One of the most important aspects in the development of the coupon scale test procedure for damage tolerance investigation was to choose a representative impact test setup. There are two important system characteristics identified in the design guideline [38] which must be taken into account to capture the correct structural responses inline with global impact scenario—(1) geometry of the impactor, and (2) appropriate boundary conditions (BCs) for the samples in the test setup.

**Figure 4:**
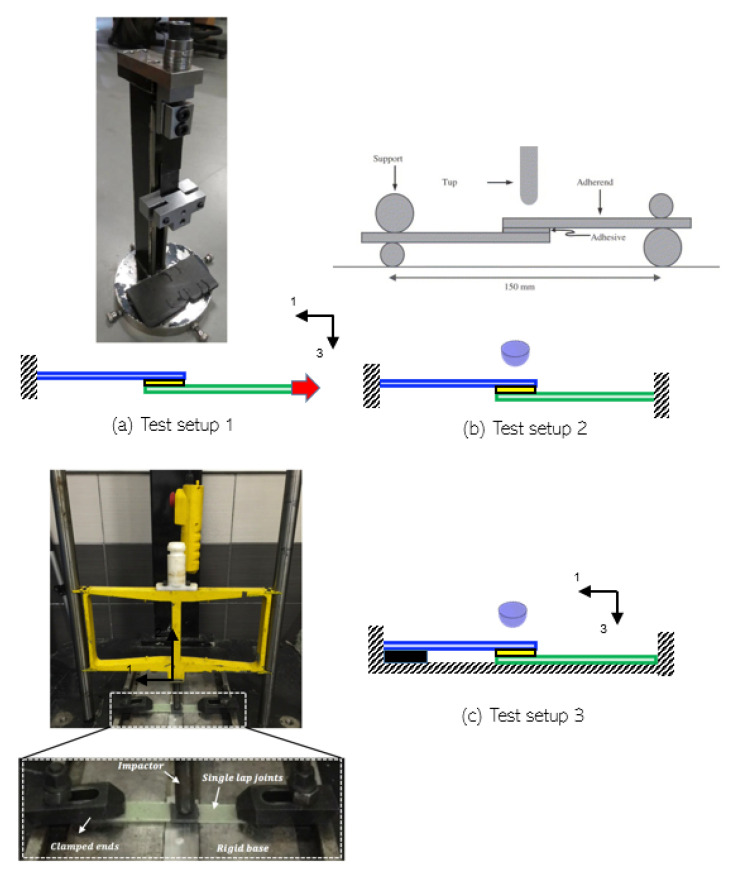
Different test setups used for testing adhesive joints under impact loads [51,56,59].

The impactor is assumed spherical and highly stiff in the study given that the impactor in the global impact scenario corresponds to a surrounding structure, such as the tower [9]. Moreover, for selecting a relevant boundary condition and a suitable test setup for our study, there were two main criteria that were to be satisfied—(1) the direction of impact loading must be in the through-the-thickness direction of the adhesive joint, and (2) the impact loads should cause localized impact damage at the adhesive zone. Additionally, the test setup must not allow any global deflection induced membrane stresses in the composite substrates since we intend to isolate the role of bondline thickness on the damage tolerance of joints. Eradicating any possibility of impact-induced membrane stresses in the substrates aids in consistent comparison of results for joints with different bondline thickness. The issues with the boundary conditions are discussed below and merits and demerits for different test setups used in the literature and their suitability for our application are argued. There are primarily three test setups identified in the literature for impact loads on single lap adhesive joints (see Figure 4a–c):

(1) Test setup 1: Under this test setup, boundary conditions include one end of the joint being rigidly fixed, whereas the other end is unconstrained in the direction of force (F) (see Figure 4a). An impactor is allowed to drop in such a way that it impacts the lower end of the fixture attached to the sample [44,45] (see Figure 4a). In other words, impact forces are imposed in the in-plane direction of the single lap joints in order to break the adhesive bond. This test setup is also referred to as in-plane dynamic testing with high strain rate, where the substrates of the joints are dominantly exposed to membrane forces. The test setup has been used extensively in the literature sources by [44,46,47,48,49,50] to investigate impact assessment on single lap joint. However, this test setup is contrary to the global impact scenario in our study applied to the leading edge of a wind turbine blade. Hence, this test setup was not considered suitable for the current study.

(2) Test setup 2: This test setup includes both ends of the sample being rigidly fixed, and a drop weight impact machine is used to apply impact energy in through the thickness direction of the sample (see Figure 4b). This configuration of loads is explicitly referred to as the transverse impact loading condition in the literature (perpendicular to the reinforcement direction) and has been utilized in [51,52,53,54,55]. The dominant mode of failure is governed by the membrane effects in the substrates, global deflection of the sample under impact-induced bending, as well as localized deformations. Hence, mixed loading and failure modes will likely cause a severe inconsistency when comparing three different bondline thicknesses. Therefore, this test setup was not considered suitable for the study.

(3) Test setup 3: The configuration of loads and BCs for this test setup is similar to Test setup 2, however, a rigid support base is present below the samples along with rigidly clamped ends (Figure 4c). In this way, the local impact damage on the adhesive joint is isolated by eliminating any significant membrane forces and global bending deformation. Furthermore, the flat rigid plate also satisfies the geometric stiffness inherently present in the blade system due to the curvature of the leading edge section. Hence, this test setup enables a consistent comparison of localized impact-induced effects on adhesive joints between different bondline thicknesses. The test setup has also been explored in the literature [56,57,58] for impact testing on adhesive joint, and was judged as the most suitable configuration for the present study.

### 2.3. Choice of Impact Energy at Coupon Scale

**Figure 5:**
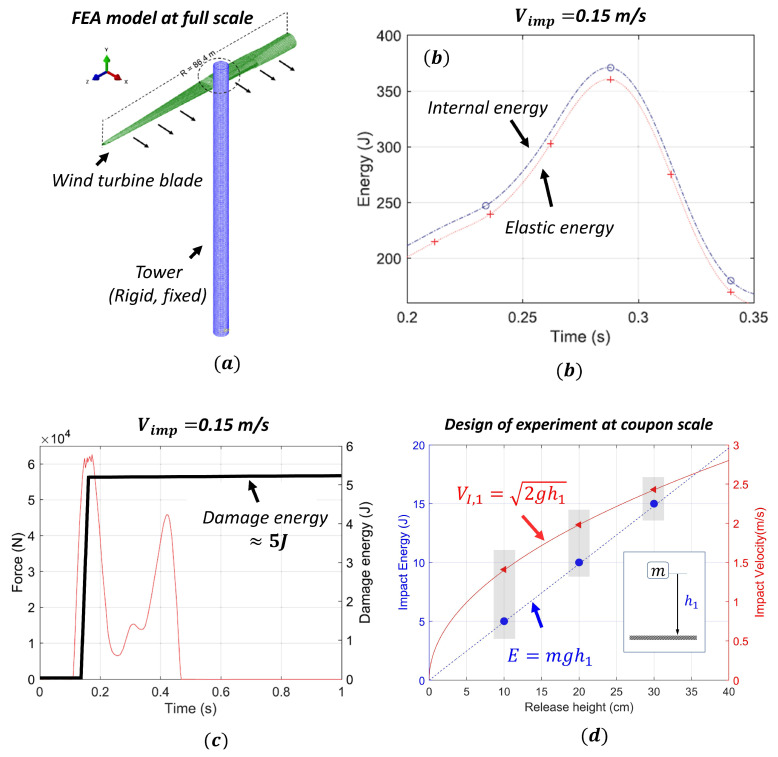
(**a**) Full scale Finite Element Model (FEA) model from [2], (**b**) comparison of internal energy and elastic energy in the blade [2], (**c**) magnitude of damage energy developed in full scale blade [2], and (**d**) coupon scale impact parameters.

The final design aspect of test procedure was to choose a representative range of impact energies to be utilized at the coupon scale for impact testing. The main idea was to keep the energies associated with damages at a full scale (damage energy) consistent with energies associated with damages at a coupon scale for damage tolerance investigations. The range of damage energy obtained in the blade due to impact during installation is obtained from our previous work [2], where we performed a finite element analysis (FEA) at full scale (Figure 5a). Principally, during lifting and under normal scenarios (ignoring accidental scenarios), the blade can impact the surrounding structures with low velocity (expected range 0–0.5 m/s); nevertheless, impact energy during the collision of the blade is still high since a blade can weigh several tons (e.g., an open source 10 MW blade [60] used for full scale investigation in [2] weighs around 41 tons). In the investigation, it was found that only a fraction of the impact energy was absorbed in the blade as damage, while the majority was dissipated as recoverable elastic strain energy by means of rigid-body motions. For instance, Figure 5b presents energy absorption results for a specific case where the blade impacts the tower with an impact velocity of 0.15 m/s. The figure shows curves for internal energy and elastic energy developed in the blade due to impact. As seen from the figure, these curves do not overlap, which implies some energy is absorbed as damage referred to as damage energy, which is found in the order of 5 J (Figure 5c). This magnitude of damage energy at full scale investigation forms the basis for the choice of initial impact energy in our experiment at coupon scale. In this study, we considered 5 J–15 J of impact energy for investigation which lies in the range of damage energy that can be expected due to impact during installation. Considering a classical drop weight impactor test setup for coupon scale investigation, the desired impact energy on the sample can be generated by releasing an impactor with mass (*m*) from a release height ($h_{1}$) onto the specimen. Figure 5d presents the desired release height as 10 cm, 20 cm, and 30 cm to achieve an impact energy of 5 J, 10 J, and 15 J using an impactor that has a mass of 5.1 kg. Furthermore, utilizing the impact kinematics, the impact velocity ($V_{I,1}=\sqrt{2gh_{1}}$) is obtained as 1.41 m/s, 1.98 m/s and 2.43 m/s, respectively.

## 3. Materials and Methods

### 3.1. Preparation of Composite Substrates

Experimental investigations are performed on single lap joints made with GFRP substrates, and joined together by Araldite 2015-1 adhesive. The Araldite 2015-1 [61] is a two component epoxy-based structural adhesive from Huntsman and has been widely used for bonding GFRP components in the wind turbine blade and aerospace industry. Furthermore, the standards from ASTM D1002-01 [62] and ASTM D2093-03 [63] are used for deciding the correct dimensions and methods for single lap joint (SLJ) preparation for obtaining adhesive joints of the highest quality.

**Figure 6:**
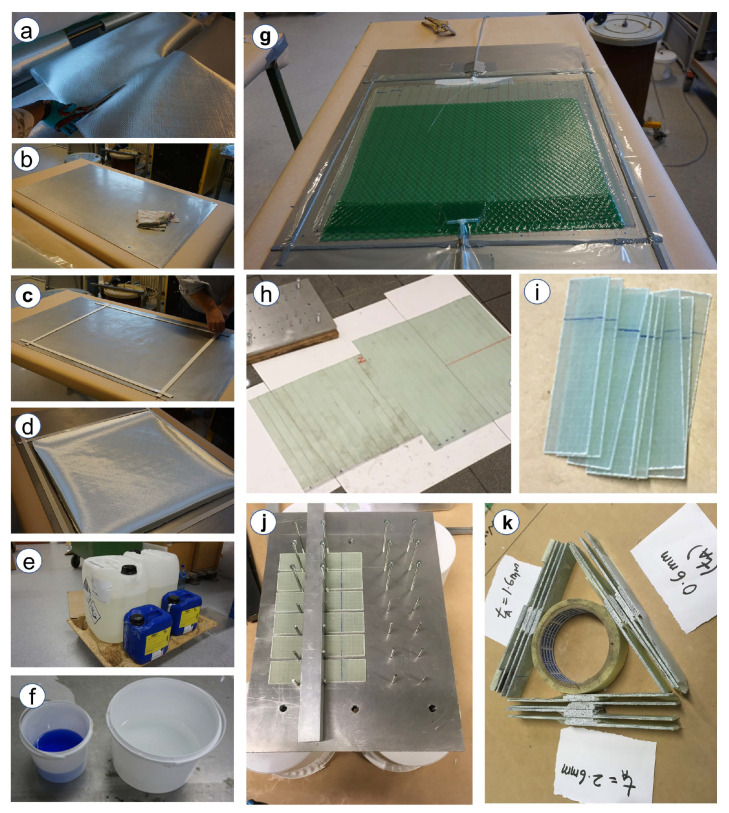
Step-by-step procedure (**a**–**k**) followed in experiment for preparing laminates and adhesive joints.

GFRP laminates are manufactured using vacuum infusion process, a standard manufacturing procedure followed in the wind turbine blade industry. The procedure for preparation of joints are summarized in Figure 6 and are also briefly explained in the sequence below. First, unidirectional (UD) and biaxial reinforcements from Saertex made of 3B’s HiPer-tex W2020 glass fibers are cut in square sheets of size 60 cm × 60 cm (Figure 5a) and are stacked on the mould to obtain a layup plan ${\left[+45/-45/0\right]}_{s}$. Note that before stacking the reinforcements, the stacking mould is unstained with acetone and a release agent is used (Figure 6b). Then, the sealing tapes are applied on the mould (Figure 6c), and the desired sequence of reinforcement along with peel ply and flow meshes are stacked (Figure 6d). Momentive EPIKOTE-MGS135 epoxy resin and EPIKURE-MGS137 curing agent are mixed in a 3:1 ratio, and the mixture is used as the resin (Figure 6e,f). The mixed resin is then kept in the degassing chamber for removing any trapped air bubbles. Furthermore, the whole system is vacuum infused at a pressure of 0.8 bar (Figure 6g) and the prepared laminate has a final thickness of 2.2±2% mm. The curing cycle of the laminate included keeping the prepared laminates at room temperature for 24 h followed by their post curing in the oven at 80 ${}^{\circ }$C for another 15 h. The prepared laminates are further cut into desired dimensions of size 100 mm × 25 mm for their use as substrates in the SLJs (Figure 6h,i).

### 3.2. Preparation of Single Lap Adhesive Joints

**Figure 7:**
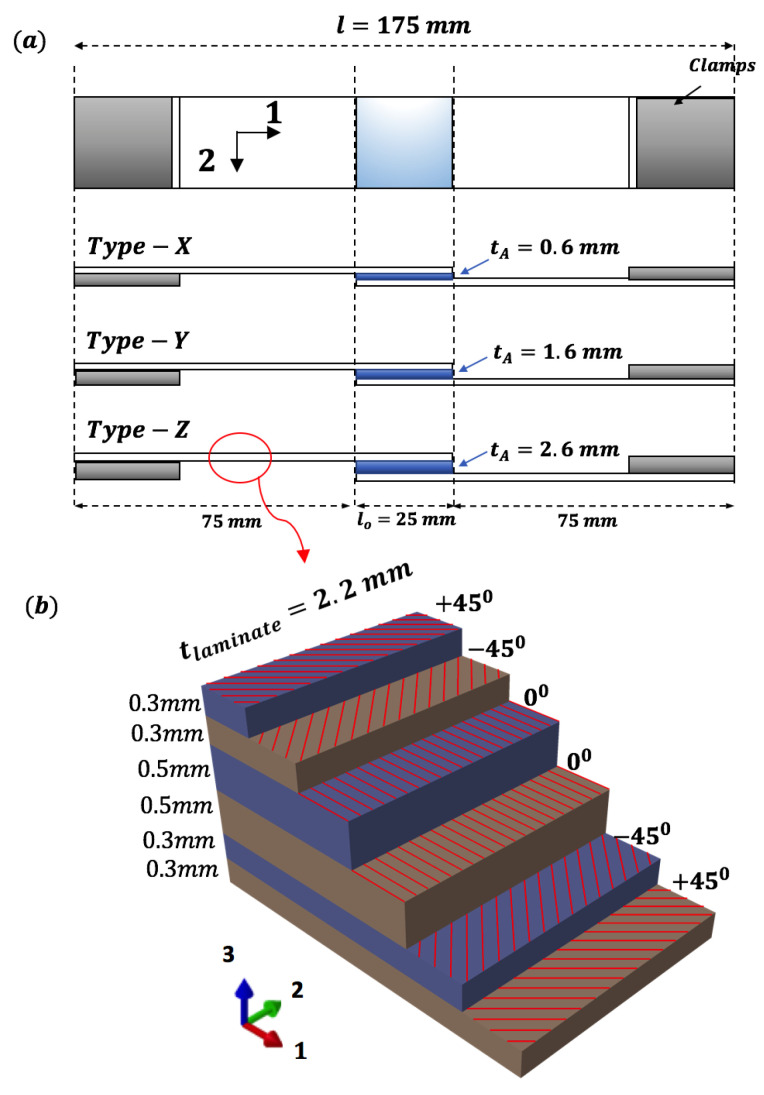
(**a**) Configuration of prepared adhesive joints and (**b**) layup of top and bottom substrate.

For preparing single lap joints, the components of Araldite 2015-1 adhesive, i.e., resin and hardener, are mixed manually in the ratio of 1:1 parts by weight. Before applying adhesive to the substrate, their surfaces are cleaned with acetone. Then, the prepared adhesive is manually applied over the overlap length of substrate towards the rough surface and a specially constructed setup for obtaining a controlled thickness of adhesive joints is utilized (Figure 6j). The joints are then kept at room temperature for 8 h followed by post curing in the oven at 40 ${}^{\circ }$C for another 16 h. The excessive adhesive along the side of the joints are also removed using manual grinding. Three different joints with varying adhesive thicknesses ($t_{a}$) are prepared and numbered with following nomenclature: Type-X joints with a post-cured thickness of adhesive as ($t_{a}^{x}=0.6$ mm), Type-Y with a post-cured thickness of adhesive as ($t_{a}^{y}=1.6$ mm), and Type-Z with a post-cured thickness of adhesive as ($t_{a}^{z}=2.6$ mm) (Figure 6k). The relative thickness of layers and adhesive correspond to configurations used in the leading edge of a wind turbine blade. Figure 7a presents the configuration and dimensions of prepared adhesive joints. The total length of the joints is 175 mm, with an overlap length of 25 mm. The stacking sequence of substrate ${\left[+45/-45/0\right]}_{s}$ is also presented in Figure 7b. For each bondline thickness, there are 16 samples prepared, where 4 samples are tested under a lap shear test for measuring the intact failure load, and the remaining samples are used for damage tolerance investigation.

### 3.3. Lap Shear Tests of Intact Samples

**Figure 8:**
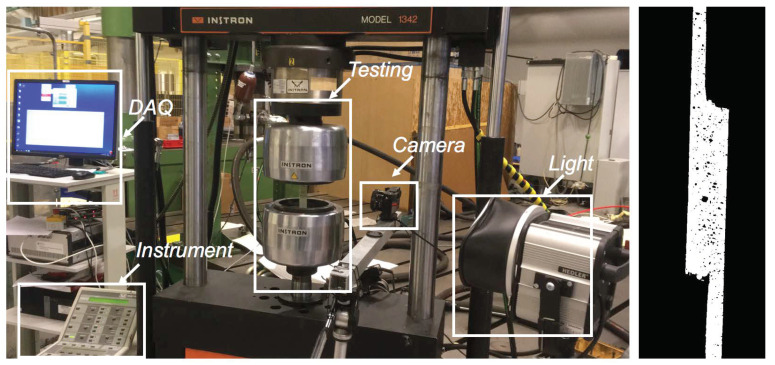
Setup for testing adhesive joints under lap shear (**left**) and through the thickness surface prepared for failure analysis (**right**).

The prepared single lap joints are tested in a universal testing machine (UTM) Instron model 1342, at a displacement rate of 1 mm/min. Figure 8a presents the test setup, where components such as data acquisition system (DAQ), operating instrument, camera, light, and samples are shown. In addition, the initial configuration of the joint along the thickness direction, which is used as a surface for image analysis of the failure process is shown in Figure 8b. The maximum force observed by the UTM machine is considered as the failure load of the intact samples. Note that all tests are carried out at room temperature ranging between 23 ${}^{\circ }$C to 25 ${}^{\circ }$C.

### 3.4. Impact Testing of Single Lap Joints (SLJs)

**Figure 9:**
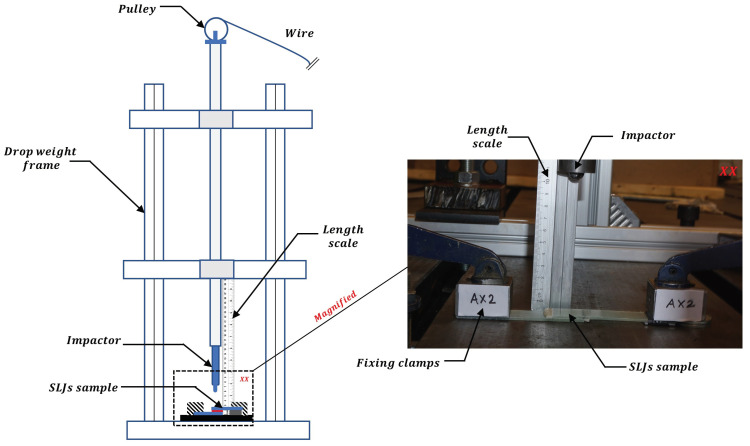
Test setup constructed for impact testing on single lap adhesive joints.

A special dedicated fixture for testing joints under impact loads is constructed for this study to replicate the boundary condition (see Figure 9). The test setup frame is assembled using strut profiles and connections from Bosch Rexroth AG [64], and the base of the setup is rigidly fixed to a steel base. A high-grade hollow steel cylinder is rigidly connected to the center of the frame (Figure 9), which acts as the guiding chamber for the impactor to fall on the sample. The impactor is a rigid steel cylinder having a mass of 5.1 kg and has an impactor diameter ($\phi_{d}$) of 12.7 mm. One end of the impactor is attached with the fibre rope which runs through the pulley attached to the top of the hollow cylinder, and the rope is connected with the winch which controls the height of the impactor. A length scale is also attached with the setup which measures the release height and the rebound height of the impactor. In this way, the impact energies in the test are controlled.

A rigid base is used to hold the samples for impact, and the samples are clamped at the ends along with suitable tabs placed on one end. A camera capable of capturing the entire impact event is used during the test, and the recorded video is further utilized to analyze the impact kinematics as discussed in the next section. A magnified image of the sample is shown in Figure 9, which corresponds to impact energy of 5 J and release height of approximately 10 cm. Furthermore, for applying impact energy of 10 J and 15 J, the height of the impactor corresponds to approximately 20 cm and 30 cm, respectively (see Figure 5d). Note that the samples are properly numbered in a unique nomenclature. For example, in Figure 9, the sample is numbered as ‘AX2’. Here, the first letter of the nomenclature ‘A’ corresponds to an impact energy of 5 J (B will correspond to impact energy of 10 J, and C will correspond to impact energy of 15 J), the second letter ‘X’ of the nomenclature corresponds to bondline thickness of 0.6 mm (‘Y’ will correspond to bondline thickness of 1.6 mm, ‘Z’ will correspond to bondline thickness of 2.6 mm), and the third letter of the nomenclature corresponds to the sample number.

### Analysis of Impact Kinematics

**Figure 10:**
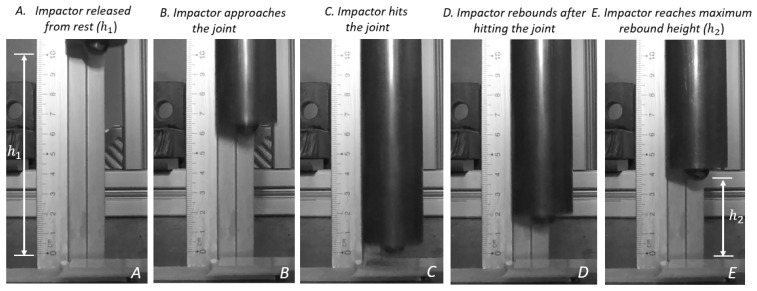
Different stages of impact event recorded by camera.

Impact kinematics were analyzed to describe the absorbed and elastic energies during the impact event. For this, we described the impact on adhesive joints into five sequential stages (Figure 10):

Stage (A) Impactor released from rest at height ($h_{1}$);

Stage (B) Impactor approaches the joint;

Stage (C) Impactor hits the joint with velocity ($V_{I,1}$);

Stage (D) Impactor rebounds after hitting the joint with rebound velocity ($V_{I,2}$);

Stage (E) Impactor reaches the maximum rebound height ($h_{2}$).

The video recording from the camera was post processed to obtain the release height ($h_{1}$) and rebound height ($h_{2}$) for all the cases. The coefficient of restitution between the impacting body (the steel impactor and the composite joint in this study) is defined by [65,66]:

(1) $$COR=\frac{V_{J,2}-V_{I,2}}{V_{I,1}-V_{J,1}}.$$

Considering that the joint is stationary before and after impact ($V_{J,1}=V_{J,2}=0$) and ignoring the air drag resistance, COR can now be defined as:

(2) $$COR=\frac{-V_{I,2}}{V_{I,1}}.$$

Furthermore, the velocity of impact and rebound velocity can be obtained as [65,66] (negative sign of $V_{I,1}$ indicates a coordinate system, where positive is considered in the upward direction):

(3) $$V_{I,1}=-\sqrt{2gh_{1}}\;\mathrm{and}\;V_{I,2}=\sqrt{2gh_{2}}.$$

Finally, COR is defined in terms of release ($h_{1}$) and rebound height ($h_{2}$) as [65,66]:

(4) $$COR=\frac{-V_{I,2}}{V_{I,1}}=\sqrt{\frac{h_{2}}{h_{1}}}.$$

Note that COR can vary between 0 and 1 for elastic plastic impact, where COR = 1 corresponds to an ideal elastic impact, and COR = 0 corresponds to a perfectly plastic impact [65,66,67]. In addition, it has been found in the literature [65,66,67] that COR depends upon impact velocity as well as size and shape of the impacting surface and is also related to the energy loss percentage (%ΔD) in the system [66] during the impact as:

(5) $$\%\Delta D=100(1-COR^{2})$$

**Figure 11:**
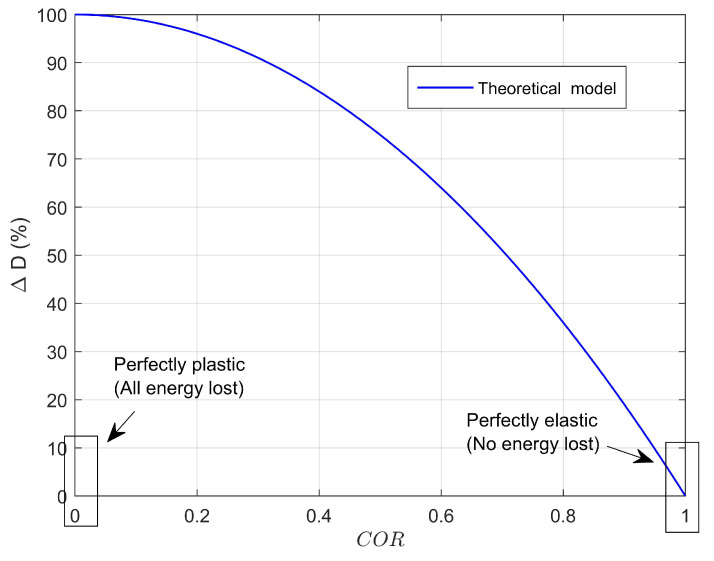
Description of impact kinematics: Theoretical %ΔD−COR curve.

A %ΔD−COR theoretical curve is presented in Figure 11, where it can be seen that the lower the value of COR, the larger the energy loss percentage %ΔD in the system during the impact. Note that the elastic part of the impact event will cause a rebound of the impactor and is associated with energy stored due to elastic deformation [68]. On the other hand, the inelastic part of the impact will include energy losses in the system due to the conversion of kinetic energy of impact into thermal energy, sound, as well as localized deformation and damage of the softer material. In this study, we do not isolate the inelastic part of the impact and assume the energy loss during impact is absorbed by the specimens [68].

### 3.5. Post Impact Assessment

The damaged adhesive joints are further tested under lap shear at a displacement rate of 1 mm/min. The displacement-time history is recorded and the largest load recorded by the UTM machine is considered as the failure load of the damaged samples.

## 4. Results and Discussion

### 4.1. Assessment of Intact Failure Loads of Adhesive Joints with Different Bondline Thicknesses

**Figure 12:**
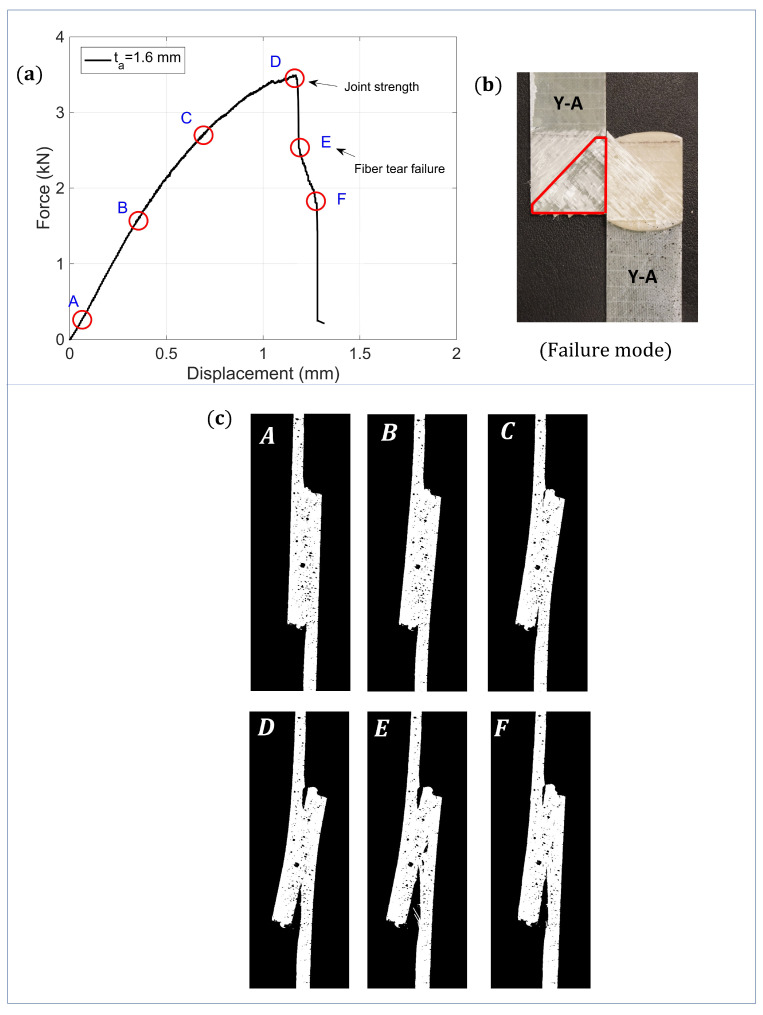
Tensile lap shear test results for intact joints: (**a**) Force-displacement history, (**b**) dominant failure mode, and (**c**) state of joint at different points A–H along the force-displacement history.

Figure 12a,b present the intact load displacement history and failure mode respectively for Type-Y samples ($t_{a}=1.6$ mm) subjected to tensile lap shear tests. The figure shows that the failure load of the sample is close to 3.5 kN, whereas the maximum failure displacement is around 1.35 mm. The failure mode is obtained in the form of fiber tear failure and partial delamination of the first ply of the bottom-most adherend at the adhesive-adherend interface. This is generally due to the fact that composite adherend’s interlaminar tensile strength is lower than the peel strength of the structural adhesive, thereby causing a delamination of the bottommost ply. This failure mode is also consistent with the results obtained in the literature for SLJs made with GFRP substrates.

**Figure 13:**
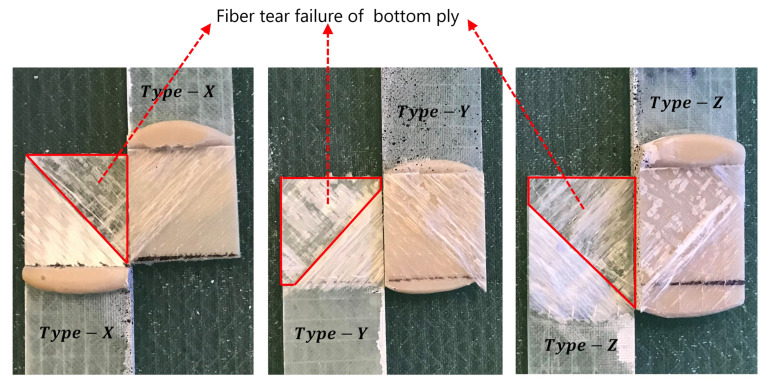
Failure mode subjected to tensile lap shear for joints with bondline thicknesses (0.6 mm, 1.6 mm, and 2.6 mm).

Figure 12a is also marked with different label pointers on the force-displacement curves, where each labeled point represents the deformation state of the sample at different time frames captured using a camera and presented in Figure 12c. It can be seen that the load curve exhibits linear behavior with increased displacement till point B. Furthermore, due to increased bending moment at increased displacement, the joints are exposed to both tensile as well as bending mode of deformation (see state C,D). At point D, the maximum load is reached, and at this point, partial delamination of the bottom most ply has completely occurred at the adhesive-adherend interface due to high peel forces. This partial delamination further progresses into fiber tear failure at E, finally causing complete failure of the joint at F. The response behavior and failure mode is consistent for all the joint-types and for all the tested samples under tensile lap shear. Figure 13 summarizes the failure mode obtained under tensile lap shear tests for all the three different bondline thicknesses ($t_{a}$ = 0.6 mm, 1.6 mm, and 2.6 mm) and for one representative sample. As seen from the figure, the dominant failure modes for all the cases include fiber tear failure and partial delamination of the first ply of the bottom-most adherend at the adhesive-adherend interface.

**Figure 14:**
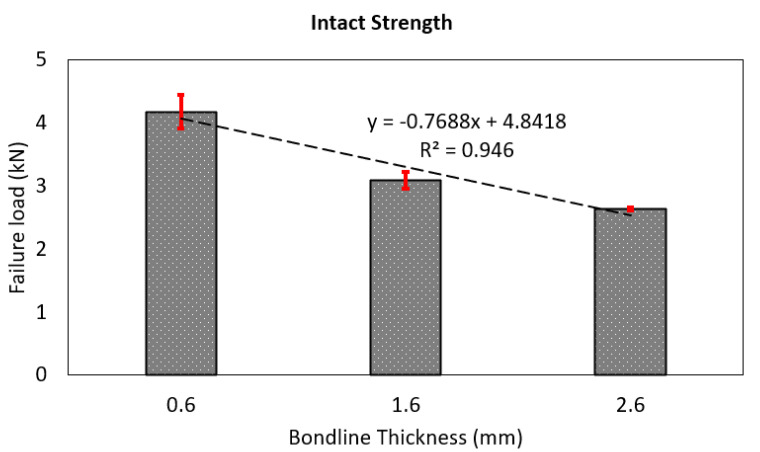
Comparison of intact failure loads with varying bondline thicknesses.

Figure 14 further compares the failure load for the joints with different bondline thicknesses under a tensile lap shear test. It is seen from the figure that the failure load is the highest for the joints with the least bondline thickness ($t_{a}=0.6$ mm), whereas the failure load is the lowest for the joint with the largest bondline thickness ($t_{a}=2.6$ mm). This is due to large bending moment developed due to eccentricity of the load application, and this effect increases with increasing bondline thickness. Overall, the failure load of the single lap joint reduces with increasing bondline thickness. This result is in agreement with the observation made in the published literature on the failure load of GFRP-based single lap joints subjected to tensile lap shear load [8]. A line of best fit is also drawn to confirm this correlation, which showed a negative slope and has an $R^{2}$ value close to 95% confirming our overall observations.

### 4.2. Impact Damage Assessment and Impact Kinematics

**Figure 15:**
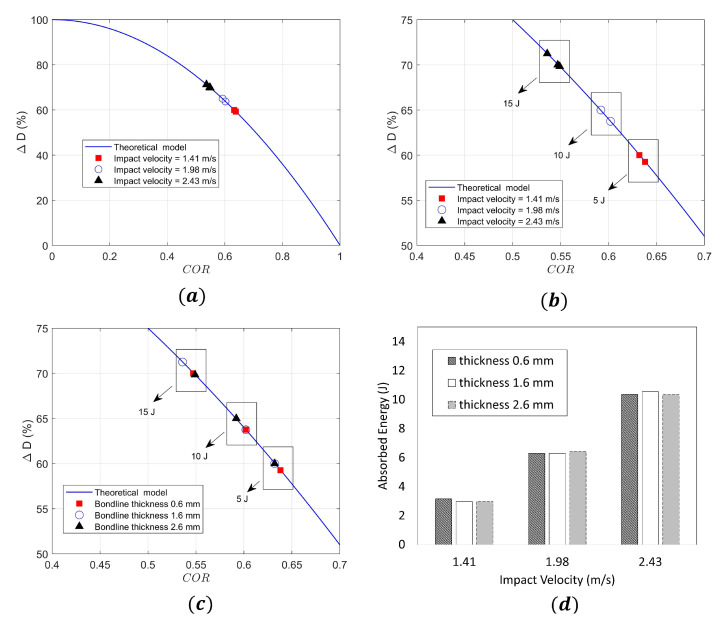
Description of impact kinematics: (**a**) %ΔD−COR curves for different impact velocities, (**b**) magnified image showing variation of COR and %ΔD, (**c**) %ΔD−COR curves for different bondline thicknesses, and (**d**) absorbed energy in the specimens for different impact velocities and bondline thicknesses.

In this section, impact kinematics describing elastic/absorbed energies together with damage assessment results under impact loads for the joints will be discussed. Figure 15a,b present the results for the impact kinematics where the %ΔD−COR values from the experiment are presented for impact on joints with varying impact velocities and associated impact energies. The experimental values obtained are also overlapped with theoretical %ΔD−COR curves in the figure.

It can be seen from the figure that the COR varies between 0.53 to 0.63 for all the cases, which implies that not all the kinetic energy of impact is absorbed in the specimen, thus implying that the impact kinematics belongs to an elastic plastic regime. Furthermore, the COR is found to decrease with increasing impact velocities which is similar to the trend observed in the literature [66]. This means that for higher impact energies, there are larger percentage energy losses in the system during impact. For instance, the average percentage energy loss in the system for the case with an impact energy of 5 J is in the order of 60%, whereas average percentage energy loss in the system for 15 J impact energy is close to 70% (see Figure 15b). Figure 15c further presents the measured %ΔD−COR values from the experiment for the impact of joints, now classified according to different bondline thicknesses. It can be seen from the figure, that for a given impact energy, there is a weaker dependence of COR on the bondline thickness compared to the impact velocity discussed before. This implies that for a given impact energy, COR and percentage of energy loss due to impact in the system do not vary significantly with bondline thicknesses. This can also be seen from Figure 15c,d, where for a given impact energy, the range of energy absorbed by the sample is similar for joints with varying bondline thicknesses. For instance, around 3 J of impact energy is absorbed by joints with bondline thicknesses of 0.6 mm, 1.6 mm, and 2.6 mm associated with an impact velocity of 1.41 m/s (5 J); 6 J of impact energy is absorbed for the impact velocity of 1.98 m/s (10 J), and 10.5 J of impact energy is absorbed for an impact velocity of 2.43 m/s (15 J) (Figure 15d).

**Figure 16:**
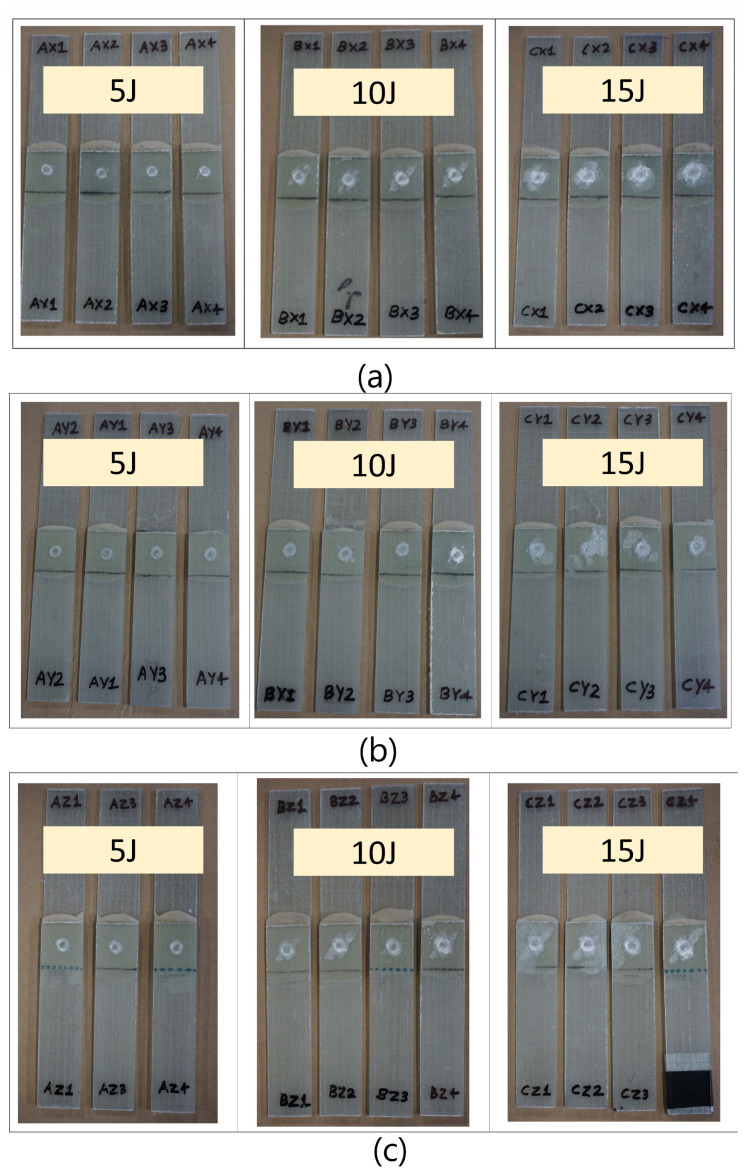
Damages in the joints subjected to different impact energies of 5 J, 10 J, and 15 J for bondline thickness: (**a**) 0.6 mm, (**b**) 1.6 mm, and (**c**) 2.6 mm.

**Figure 17:**
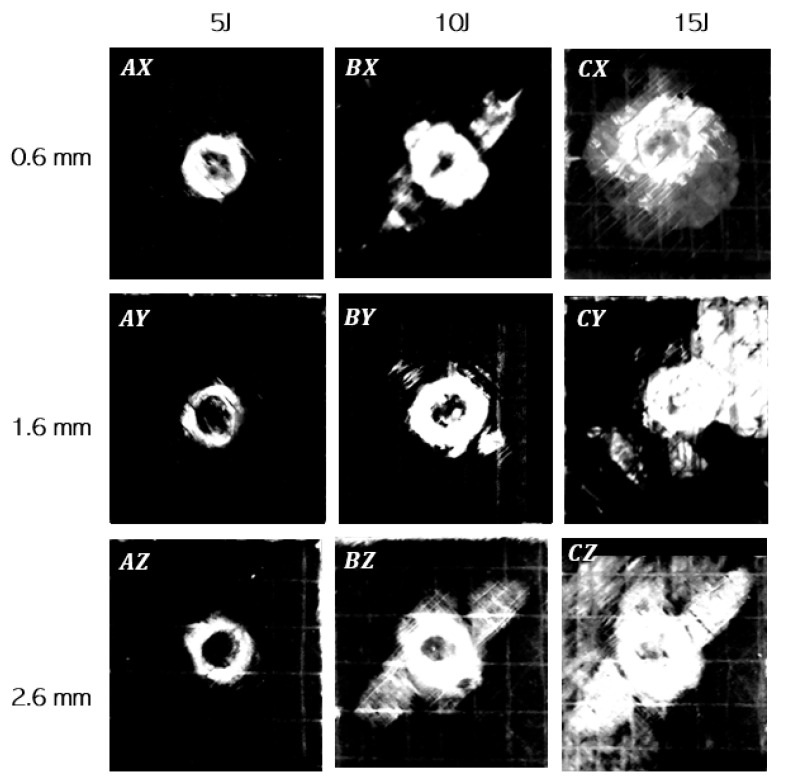
Projected damage area obtained in the joints with bondline thickness (0.6 mm, 1.6 mm, and 2.6 mm) and subjected to impact energy (5 J, 10 J, and 15 J).

**Figure 18:**
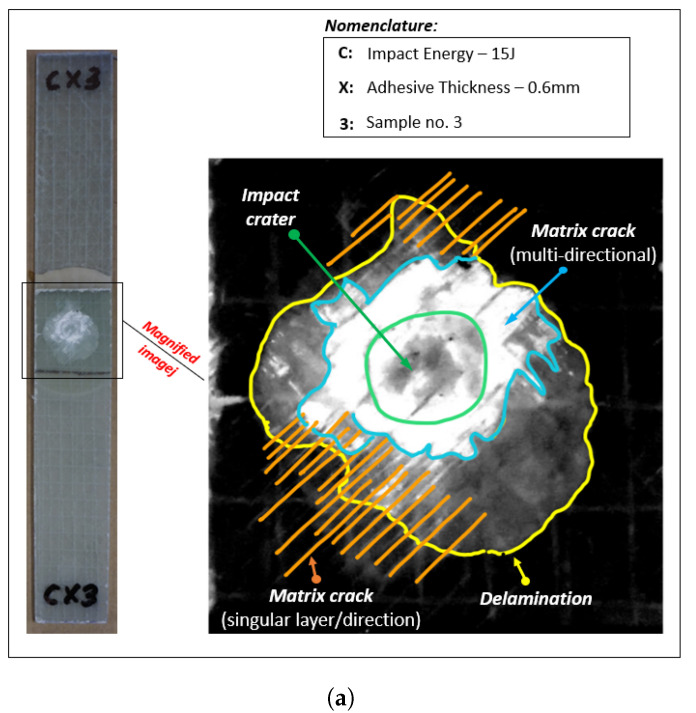

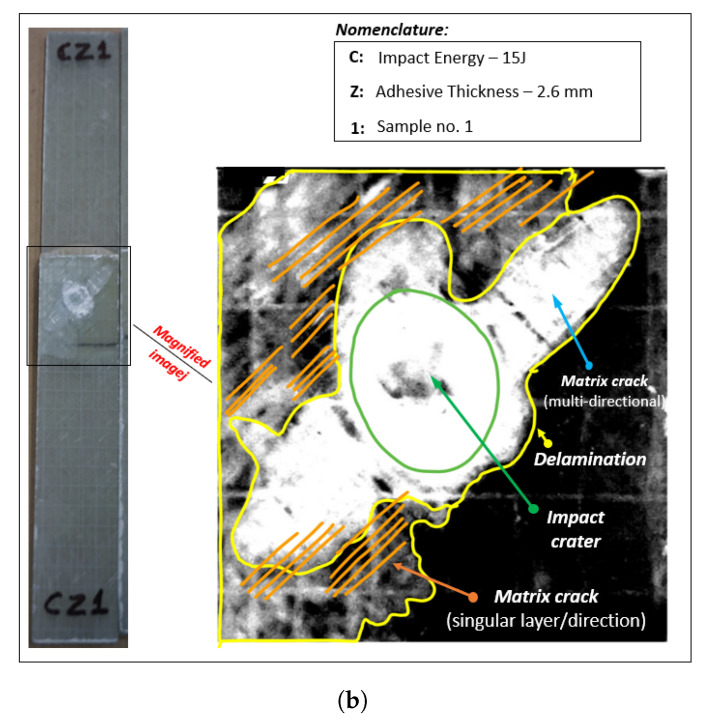
Different failure modes in the joints subjected to impact energy of 15 J for bondline thickness of (**a**) 0.6 mm and (**b**) 1.6 mm.

Figure 16a–c present the damages obtained in the specimens due to impact loads. The damages shown here are for all the joints with bondline thicknesses ($t_{a}=0.6$ mm, $t_{a}=1.6$ mm, and $t_{a}=2.6$ mm) respectively subjected to three different impact energies (5 J, 10 J, and 15 J). Note that we used an open-source image processing software, imagej [69], for quantifying the projected damage area due to impact loads (see Figure 17 and Figure 18). The main focus of the damage assessment was not to quantify the damages through the thickness of the sample in detail, and we restricted our investigation to the overall projected area of damage.

**Figure 19:**
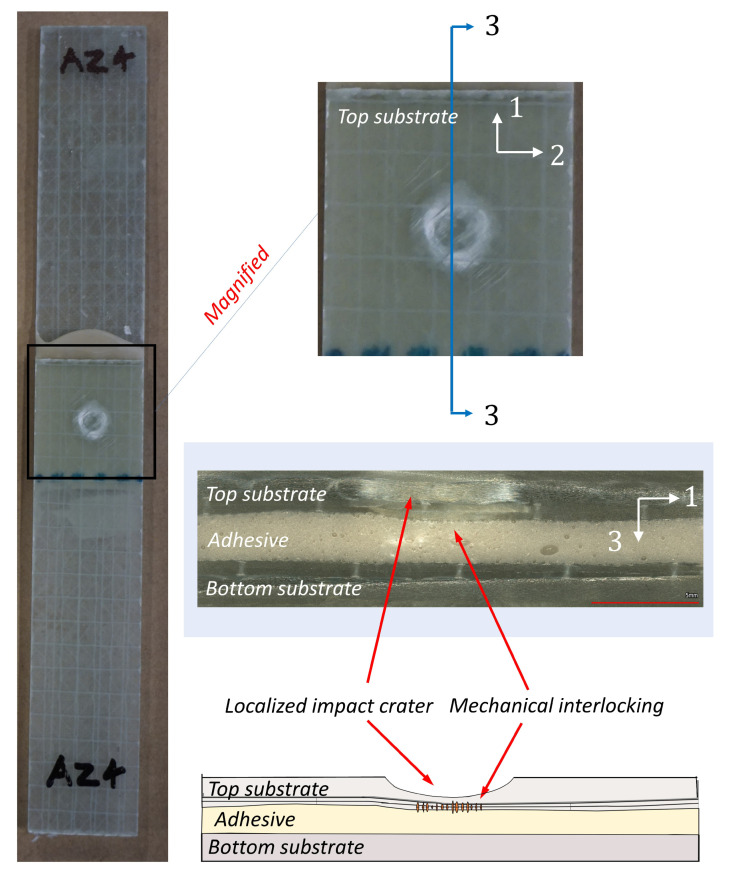
Cross-sectional scan of joint with bondline thickness of 0.6 mm subjected to 15 J of impact energy.

As seen from Figure 16 and Figure 17, the damages in the joints due to impact loads are found to be predominantly concentrated around the overlap adhesive zone, and explicitly around the impact point. However, for the largest impact energy of 15 J, the damages even extended to the joint edges for all the samples related to bondline thickness of $t_{a}\;=2.6$ mm and one sample corresponding to the bondline thickness $t_{a}$ = 1.6 mm (white area extended around the edges). These failure types and regions are critical locations of high peel stresses when subjected to tensile lap shear testing. Figure 18a,b further magnify and annotate different failure modes obtained for two different joints with varying bondline thicknesses and an impact energy of 15 J. As seen from these figures, the failure modes in the joint include matrix cracking, typical bean-shaped delamination pattern in the top composite adherend, as well as an impact-induced localized crater around the impact zone. The localized circular crater is one of the most important features found in all the joint types subjected to varying ranges of impact energies (see Figure 15, Figure 16 and Figure 17). The depth of the localized crater was found to increase with increasing impact energy and to be the most critical for thin joints. This is because for thin joints, the localized crater caused plastic deformation in the layers of the top composite adherend, which extended until the bondline interface. This localized crater along the adhesive zone has been found in the literature [56,57] to cause mechanical locking effects that prevent relative movement of the adherend during the tensile lap shear testing, thereby affecting the overall strength and stiffness of the joint. For additional clarity and representation of these interlocking effects, a cross-sectional scan of one of the thin samples with a bondline thickness of 0.6 mm (cut lengthwise across the impact crater) is presented in Figure 19. We utilized an optical microscope Keyence VR-5000 for presenting this cross-sectional analysis. Figure 19 clearly describes that the mechanical locking effect, where the adhesive layer together with the top adherend’s composite layers below the impact crater, has been found to undergo plastic deformation due to impact-induced compressive stresses.

**Figure 20:**
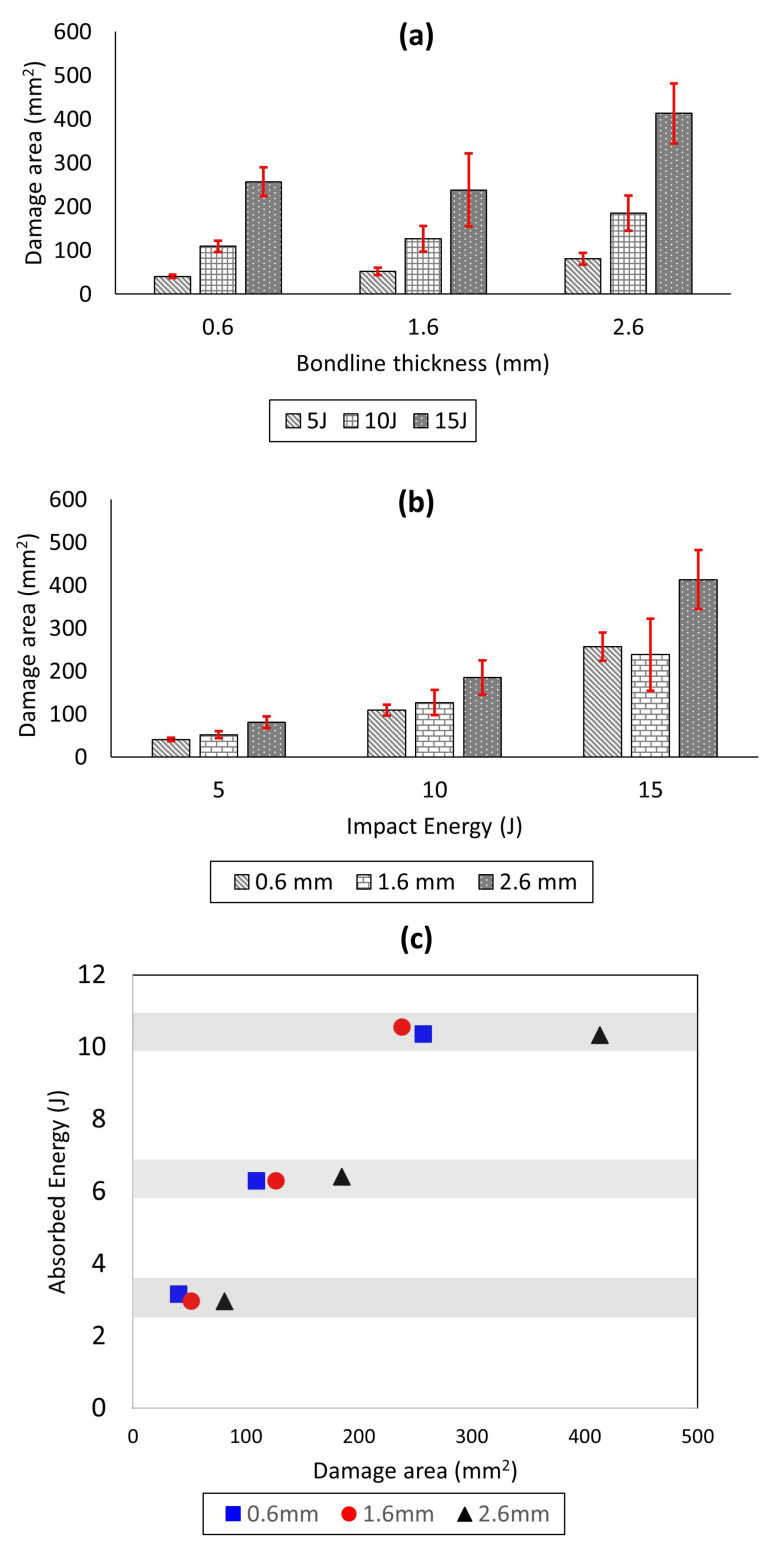
Comparison of impact-induced projected damage area in joints for different (**a**) bondline thicknesses, (**b**) impact energies, and (**c**) absorbed energies.

In addition, as seen visually from Figure 16 and Figure 17, the projected damage area on the impact surface increased with increasing impact energies for all the bondline thicknesses. This observation was further quantified in Figure 20a,b using image processing where the damage areas are presented for different bondline thicknesses ($t_{a}=0.6$ mm, $t_{a}=1.6$ mm, and $t_{a}=2.6$ mm) subjected to impact energies (5 J, 10 J, and 15 J). It is found that for any given bondline thickness, the damage area increased with the increasing impact energy, thereby validating our visual observation. These results also agree with the results presented in Figure 15a,b where COR decreased with an increasing impact energy. For instance, for the adhesive joint with $t_{a}=0.6$ mm, the damage area was around 42 mm^2^, and this increased to more than 300 mm^2^ for an impact energy of 15 J. Moreover, from Figure 20b, it was found that for a given impact energy, the adhesive joint with the thinnest bondline thickness had the least damage area, whereas the thickest joint had the largest damage area. This is an interesting observation as it was previously discussed that the absorbed energies were similar for joints with varying bondline thicknesses (see Figure 15c,d). This can also be seen from Figure 20c where the average projected damage area is plotted against absorbed energy for joints with different bondline thicknesses. The results clearly show that for the same level of absorbed energy, there is more projected damage area observed for thickest joints compared to joints with thin bondline thickness. All in all, thin joints exhibit the highest damage resistance to the projected damage area.

### 4.3. Damage Tolerance Analysis

**Figure 21:**
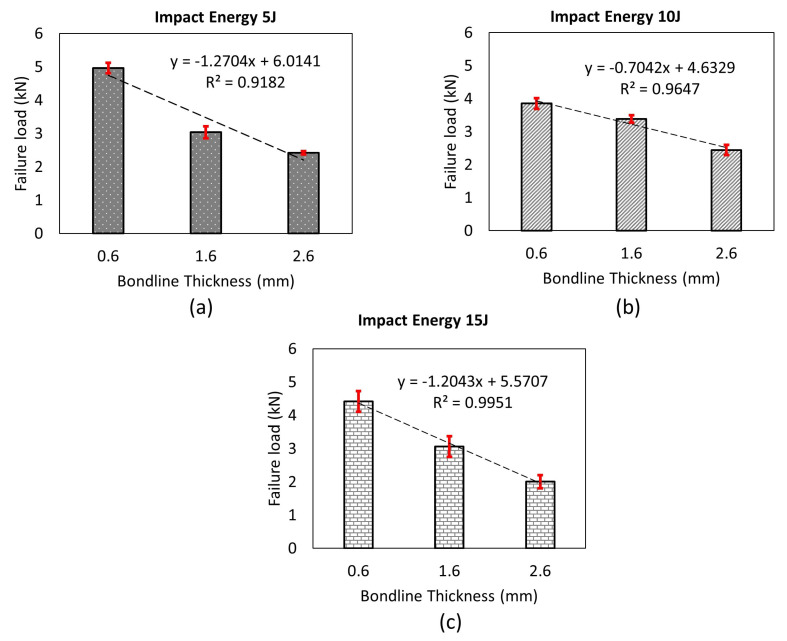
Comparison of post impact failure load of joints for different bondline thickness after being subjected to an impact energy of (**a**) 5 J, (**b**) 10 J, and (**c**) 15 J.

The damaged samples corresponding to different impact energies and different bondline thicknesses were further tested under tensile lap shear tests to measure the influence of impact loads on their failure loads. Note that the joints were tested under the same load rate of 1 mm/min which was used for measuring failure loads of intact samples. Figure 21a–c compare the failure loads for damaged samples corresponding to three different impact energies (5 J, 10 J, and 15 J) and for three different bondline thicknesses ($t_{a}=0.6$ mm, 1.6 mm, and 2.6 mm), respectively. The results show that for any given impact energy, the largest failure load belongs to the thinnest bondline thickness, and therefore the thinnest joint exhibited the highest damage tolerance. For instance, the failure load of the joint with bondline thickness of 0.6 mm subjected to 5 J of impact energy is 5 kN, 3.1 kN for bondline thickness of 1.6 mm and 2.8 kN for a bondline thickness of 2.6 mm. All in all, it was found that similar to static behavior, the best performance of the single lap joint under impact load is given by the joint having the least bondline thickness. This observation agrees with our damage assessment results discussed previously where the projected damage area was minimum for joints with the thinnest bondline thicknesses. The failure mode is also found consistent with static testing which included fiber tear failure and partial delamination of the first ply of the bottom-most adherend at the adhesive-adherend interface. Overall, the impact loads did not modify the failure modes for the joints tested under tensile lap shear testing.

**Figure 22:**
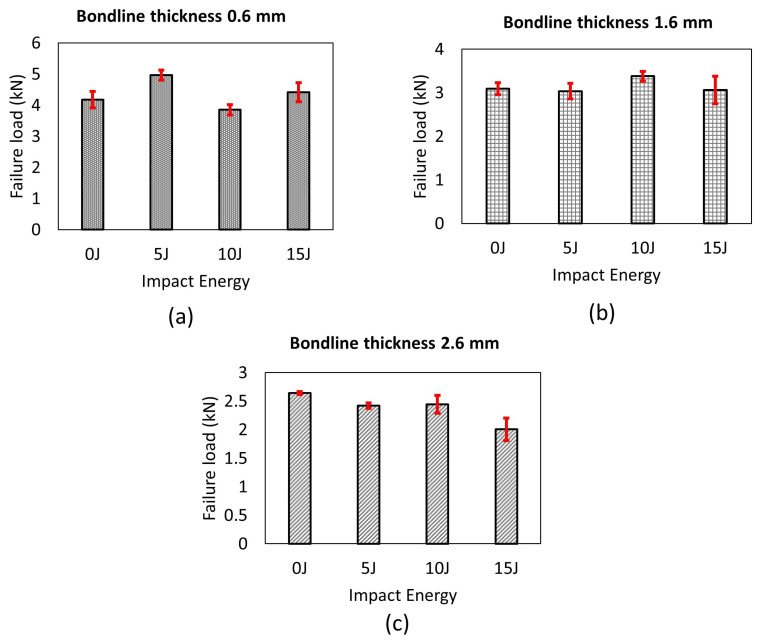
Comparison of post impact failure load of joints after being subjected to different impact energy: (**a**) Bondline thickness of 0.6 mm, (**b**) bondline thickness of 1.6 mm, and (**c**) bondline thickness of 2.6 mm.

**Figure 23:**
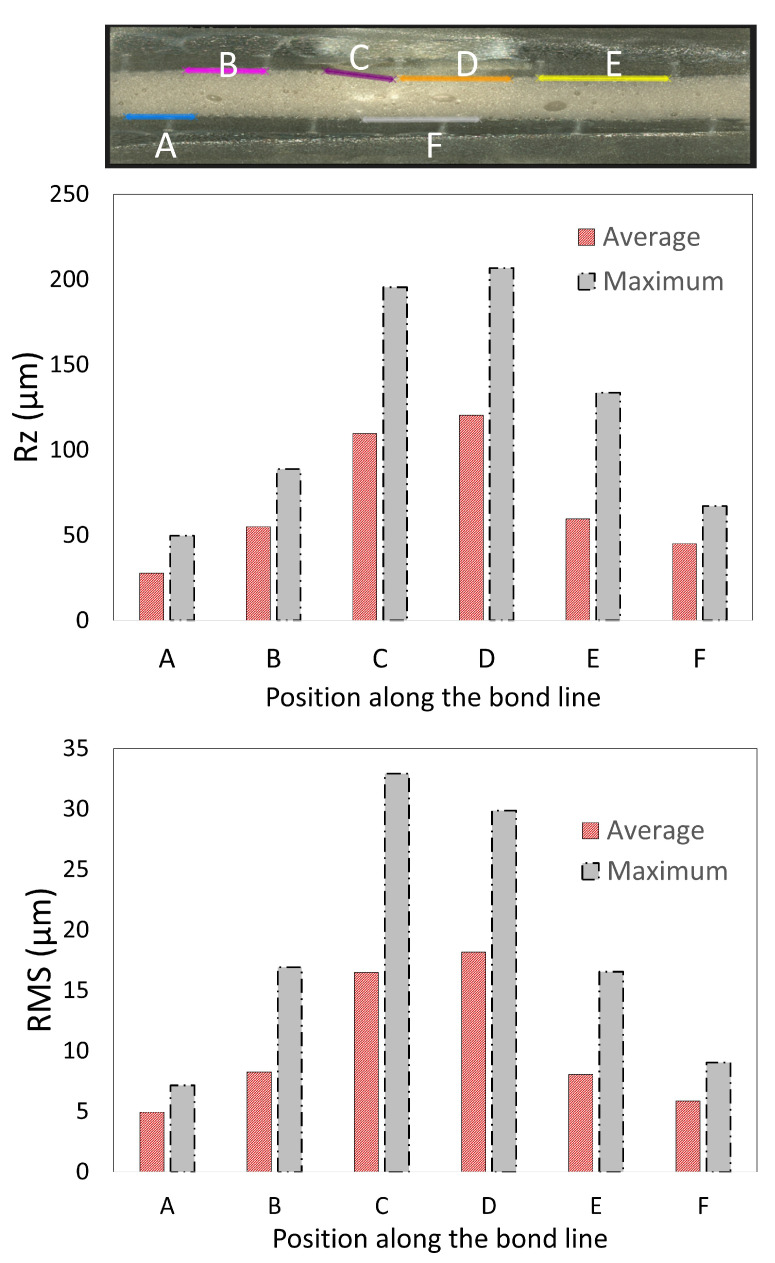
Comparison of surface roughness along the bondline interface for joint with a bondline thickness of 0.6 mm subjected to an impact energy of 15 J.

Another important finding was that for all the cases, the impact loads did not significantly reduce the failure load of joints compared to their intact values as reported in [58] for Carbon-fiber-reinforced polymers (CFRP) based adhesive joints (see Figure 22a–c). Interestingly, for some of the cases, the failure load seems to have increased by 20% compared to the intact values (see Figure 22a,b). The increase in the failure load is attributed to the impact-induced mechanical locking effects reported through the cross sectional scanning of the damaged samples (see Figure 19). The mechanical interlocking is a well-known effect that tends to increase the loading capacity of the joints by enhancing adhesion area between adhesive and substrate [70,71,72]. This enhancement could be caused by an increase in the surface roughness at the adhesive adherend interface as more fracture energy is dissipated due to increased roughness [73]. For instance, in the work from [70,71], authors added multiwall carbon nanotubes (MWCNT) to epoxy adhesive to increase the loading capacity of joint; for example, this treatment increases surface roughness and area of adhesion at the bondline interface, thereby enhancing the mechanical locking effects. In the literature [56,57], the impact induced loads on the joint have also been found to develop similar effects that tend to increase post impact loading capacity of the joints. For further quantification, we also analyzed the line surface roughness data using the optical microscope (along the plane of joint thickness) for different locations (A−F) along the adhesive adherend interface (Figure 23). We present two surface roughness parameters: Maximum height of the profile (Rz) and root mean square roughness (RMS). The results indicate that there is an increase in the surface roughness at the adhesive adherend interface (below the region of impact crater, location C, D in Figure 23), which we believe enhanced the adhesion area and increased the failure loads.

**Figure 24:**
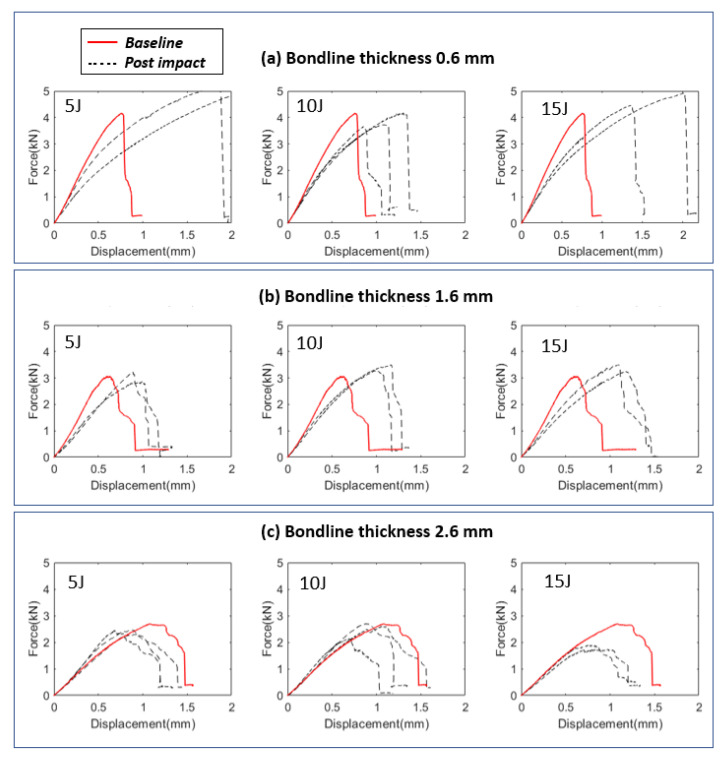
Comparison of post-impact load displacement history with intact load displacement history (baseline) for different bondline thicknesses and impact energies.

Figure 24 compares load displacement histories for intact and post-impact damaged samples subjected to lap shear tests. For thin joints, the slopes of the damaged samples were less steeper compared to the slopes of the intact joints. However, this was not the case for bondline thickness of 2.6 mm, as the slopes of the damaged samples were similar to the slopes of the intact samples, and there was a reduction in the failure load. Again, the difference in the damage tolerance behavior for different joint thicknesses was attributed to the fact that the mechanical locking effects due to the impact crater were less pronounced for the thickest joints, and the plastic deformation of composite layers did not extend till the adhesive zone. Therefore, the crater was mostly concentrated around the top adherend and damage progressed more towards the joint edges, increasing the projected damaged area. This observation implies that the projected damaged area, especially that extends towards the edges of the joint, is more detrimental to the failure load of the joint compared to the localized impact crater itself. Overall, mechanical locking effects together with projected damage area play a major role in the overall damage tolerance behavior of the adhesive joints with varying bondline thicknesses.

## 5. Conclusions

Following are the main conclusions from the study:

1. In this paper, we designed a novel coupon scale test procedure to investigate bondline thickness effects on damage tolerance of adhesive joints subjected to localized impact damages. The coupon scale test procedure represented the global impact scenario by—(1) representing the blade’s true geometrical parameters like adhesive thickness or composite thickness, joint configuration, and composite layup at coupon scale (2) using appropriate boundary conditions (BCs) to map the energies associated with damages at full scale to damages at coupon scale, and finally (3) utilizing a representative benchmark testing that would match the operational loads at the blade’s leading edge.

2. The first part of the study involved the assessment of failure load of intact adhesive joints with three different bondline thicknesses ($t_{a}=$ 0.6 mm, 1.6 mm, 2.6 mm) under tensile lap shear test. It was found that the failure load of adhesive joints is greatest for the joints with the least bondline thickness ($t_{a}=$ 0.6 mm) and the failure load reduces with increase in the bondline thickness. The dominant failure mode involved fiber tear failure and partial delamination of the first ply of the bottom-most adherend at the adhesive-adherend interface for all the cases.

3. The second part of the study included impact damage assessment and analysis of impact kinematics. Here the joints were subjected to impact energies of 5 J, 10 J, and 15 J using a test setup/boundary condition that enforces localized impact damages. It was found in the study that the impact kinematics belonged to elastic plastic regime, where COR varied between 0.53 to 0.63. Furthermore, COR reduced with increasing impact velocities, implying a higher percentage of energy loss in the system due to higher impact velocities. This observation also agreed with damage assessment where projected damage area in the joints increased with an increase in the impact energy.

4. The impact damage assessment results also revealed that for the same level of absorbed energy, there are larger projected damage areas observed for the thickest joints compared to the joints with thin bondline thicknesses. Hence, thin joints exhibit the highest damage resistance to the impact-induced projected damage area. In addition, different failure modes were identified and the localized circular impact crater was found to be critical for thin joints due to its pronounced mechanical locking effects.

5. In the last part of the study, the impact-induced damaged samples were tested under tensile lap shear tests, and post-impact failure loads were compared with the intact failure loads. It was found that for a given impact energy, the largest failure load belongs to the joint with the thinnest bondline thickness. Therefore, similar to the static behavior of adhesive joints, the best performance of the joint after impact loads was given by the joint having the least bondline thickness.

6. It was also found that for some of the thin joints, the failure load seems to have increased by 20%. The increase in the failure load was attributed to the impact-induced mechanical locking effects caused due to the localized circular impact crater at the adhesive zone. This crater caused plastic deformation in the layers of the top composite adherend as well as adhesive layer which extended until the bondline interface.

7. The study also concludes that impact damage does not influence the strength significantly, especially for the thin joints. This is an important conclusion from an engineering perspective as this result will contribute towards future damage tolerance investigations, i.e., the question of acceptable localized damage with respect to the static strength of the whole blade.
